# Microbial Growth on Dust-Loaded Filtering Materials Used for the Protection of Respiratory Tract as a Factor Affecting Filtration Efficiency

**DOI:** 10.3390/ijerph15091902

**Published:** 2018-09-01

**Authors:** Katarzyna Majchrzycka, Małgorzata Okrasa, Anita Jachowicz, Justyna Szulc, Beata Gutarowska

**Affiliations:** 1 Department of Personal Protective Equipment, Central Institute for Labour Protection—National Research Institute, Wierzbowa 48, Łódź 90-133, Poland; kamaj@ciop.lodz.pl; 2 Faculty Biotechnology and Food Science, Institute of Fermentation Technology and Microbiology, Lodz University of Technology, Wólczańska 171/173, Łódź 90-924, Poland; 801208@edu.p.lodz.pl (A.J.); justyna.szulc@p.lodz.pl (J.S.); beata.gutarowska@p.lodz.pl (B.G.)

**Keywords:** microorganisms, respiratory protective equipment, organic/inorganic dust, risk assessment, workplaces

## Abstract

This work aims at understanding the effects of various dust-loading conditions and the type of nonwovens used in the construction of FFRs on the safe use of those protective devices in situations of exposure to biological agents. The survival of microorganisms (*Escherichia coli*, *Candida albicans*, and *Aspergillus niger*) in dust-loaded polypropylene nonwovens (melt-blown, spun-bonded, and needle-punched) was experimentally determined using microbiological quantitative method (AATCC TM 100-2004). Scanning electron microscope was used to assess biofilm formation on dust-loaded filtering nonwovens. The impact of the growth of microorganisms on filtration efficiency of nonwovens was analysed based on the measurements of penetration of sodium chloride particles (size range 7–270 nm). Results showed that tested microorganisms were able to survive on dust-loaded polypropylene filtering nonwovens. The survival rate of microorganisms and penetration of nanoparticles and submicron particles depended on the type of microorganism, as well as the type and the amount of dust, which indicates that both of those factors should be considered for FFR use recommendations.

## 1. Introduction

Harmful biological agents in the work environment can have infectious, toxic, and/or allergenic effects on the human body. They pose a high risk in a number of workplaces, including those in health care, laboratory, and veterinary facilities; agriculture, forestry, food, textile, mining, and wood industry; waste collection, sorting, and processing companies; art conservation and many others [1]. Most commonly, they are present in the environment as components of bioaerosol deposited on organic dust, which can be absorbed by the respiratory tract. Among the most harmful agents that can occur in the organic dust are bacteria, fungi, allergens, endotoxins, peptidoglycans, and β(1-3)-glucans [2]. Long-term inhalation exposure to such agents may cause asthma, Sick Building Syndrome, allergic alveolitis, or Organic Dust Toxic Syndrome [3,4].

The composition and amount of organic dust to which workers are exposed depends on the type of work environment. For example, cement and composting plants have high dust concentrations in the air [5,6]. However, the composition and cytotoxicity of dust in each of these places are different. Previous studies have shown that settled dust from composting plants contain significantly more microorganisms compared to cement plants, moreover their species diversity is significantly greater [7].

The specifics of the action of biological agents relies on the fact that there is no constant relationship between their concentration or contact time and the body's response to their harmful effects. Moreover, the continuous quantitative and qualitative variability of microorganisms during the exposure of employees to these factors in the work environment has to be emphasized. Therefore, the establishment of agent-specific exposure-response relationships in workplaces is extremely complex, and hence only few health-based occupational exposure limits (OELs) were proposed for bioaerosol risk assessment [8]. As a consequence, the assessment of the occupational risk of worker exposure to biological agents is qualitative [9]. According to the EU law, i.e., 2000/54/EC Directive that outlines the principles of risk assessment, prevention, and control for biological agents [1], employers are required to assess the risks posed by biological agents. This means that they should gather information on harmful biological agents within a workplace, classify them, list the types of professional activities performed by employees that may cause exposure, and the time and extent of exposure to each agent. Any possible allergenic or toxic activity of the harmful biological agent and resulting diseases have to be determined. Employers must also analyse the registered cases of occupational diseases that were contracted by staff employed under similar work environments and the conditions of exposure to biological agents. Then proper measures to reduce the risk to the workers have to be undertaken, including elimination or substitution and exposure prevention and control. Employers should also inform and train workers and provide them with health surveillance as appropriate.

Among the measures that can minimize the consequences of health risks is the use of appropriate personal protective equipment [10]. In case of exposure to airborne biological agents, filtering facepiece respirators (FFRs) are most commonly recommended. The operating principle of such devices is the capture of bioaerosol particles from the stream of breathing air and their deposition within the respirator on the fibres of filtering nonwovens used in its construction. However, as shown in previous studies, deposited microorganisms are capable of rapid multiplication in the filtering material in conditions of high humidity (derived from exhaled air) [11]. An important factor favouring the growth of microorganisms on filtering nonwovens is organic dust, which is a source of nutrients [12,13]. Bacterial and fungal biofilms have been observed on FFRs used in high air-dust environments found in combined heat and power plants processing plant biomass [14]. These observations show that improperly used/stored FFRs can constitute a serious risk to the user, especially during prolonged or repeated use.

This potential threat should be considered in the occupational risk assessment associated with exposure to biological agents and the selection of respirators protecting against them. In addition, such factors as temperature, humidity, dust concentration, and the rate at which dust is deposited on the filtering surfaces of FFRs should be considered to ensure safe use of FFRs by workers. Unfortunately, little scientific research is available on the subject. Thus, the aim of the study was to evaluate the survival of selected microorganisms (*Escherichia coli*, *Candida albicans*, and *Aspergillus niger*) on the nonwovens used in the construction of FFRs (melt-blown, spun-bonded, needle-punched) in the presence of dust originating from work environments (cement and composting plants) and determine how joined presence of dust and microorganisms may affect the filtration efficiency of such materials.

## 2. Materials and Methods 

### 2.1. Dust

Two types of dust, A and B, were studied. Dust A, with high microbial contamination (total number of microorganisms 1.38 × 10^8^ ± 1.27 × 10^7^ CFU/g) and high carbon to nitrogen ratio (C:N = 98.64), was collected from the waste homogenization hall of composting plant (Łódź, Łódź province, Poland). Dust B, with low microbial contamination (2.10 × 10^3^ ± 1.77 × 10^2^ CFU/g) and low carbon to nitrogen ratio (C:N = 10.71), was collected from the clinker transporting conveyor hall at cement plant (Chełm, Lublin providence, Poland). Detailed microbiological, chemical, and toxicological characterization of the studied dust types has been published in [7]. Prior to testing, collected dust samples (3 of each type) were subsequently sterilized for 15 min at 115 °C and dried for 24 h at 70 °C under reduced pressure of 100 mbar in drying chamber (VD 53, Binder, Germany). An averaged specimen consisting of dust mixed at equal ratios from each sample was then prepared.

### 2.2. Filtering Nonwovens

Three types of polypropylene nonwovens typically used in the construction of FFRs were selected to test the survivability of microorganisms in the presence of dust. The characteristics of the selected nonwovens are shown in Table 1.

### 2.3. Dust Deposition in the Fibres of Filtering Nonwovens 

The samples for the study were prepared according to the methodology described in Majchrzycka et al. [15]. The filtering nonwovens were cut into circles with an area of 79 cm^2^ and subsequently placed in a dust chamber. There, air-dust mixture was passed through the nonwovens with a constant volumetric flow rate of 95 ± 5 L/min. Gravimetric method was used to establish the loading time. The amount of dust introduced to the dust chamber was adjusted so that the mass deposited on the absolute filter for 4 min was close to the mass of dust that would accumulate on the filtering layers of half-mask when used in workplace with dust concentration at an occupational exposure limit level for inhalable dust, i.e., 4 mg/m^3^ [16]. Half of the loading time (2 min) was used to achieve samples corresponding to average dust concentration at the workplace. 

Samples of filtering nonwovens were weighed before and after dust deposition and the dust content (D_p_) was calculated according to the formula:

(1) $$D_{p}=\frac{m_{p}-m\;}{m}\times 100\%$$

where: *m_p_* —mass of the sample after dust deposition (mg), *m*—mass of the sample prior to dust deposition (mg).

### 2.4. Microorganisms

Three strains from the collection of pure American Type Culture Collection (ATCC): *Escherichia coli* (ATCC 10536), *Candida albicans* (ATCC 10231), and *Aspergillus niger* (ATCC 16404) were used to assess the survival of microorganisms on dust-loaded filtering nonwovens. The strains were selected based on taxonomic variety (Gram-positive cocci, yeast, and mould) and their ability to survive in the environment by forming spores (*A. niger*) or just through vegetative cells (*E. coli* and *C. albicans*). In addition, Directive 2000/54/EC has also classified *E. coli* and *C. albicans* species in group 2 of health hazards, while *A. niger* can induce occupational allergy and infections in people with weakened immune systems [17]. Hence these species can be potentially hazardous to employees in various working environments. Bacteria and fungi were prepared by inoculating 50 mL of sterile TSB medium (Tryptic Soy Broth, Merck, Darmstadt, Germany) and yeast strains in MEB medium (Malt Extract Broth, Merck, Darmstadt, Germany), and next incubating at 37 ± 2 °C for 24–48 h. Mould inoculum was obtained by washing spores from *A. niger* culture (MEA—Malt Extract Agar, Merck, Darmstadt, Germany, 5 days at 27 ± 2 °C,) with MEB medium. Inocula at a density of 2.17 × 10^7^ CFU/mL (*C. albicans*), 2.67 × 10^7^ CFU/mL (*A. niger*), and 5.3 × 10^9^ CFU/mL (*E. coli*) were obtained. To assess the impact of the growth of microorganisms on the development of microbial biofilm and the filtration efficiency of dust-loaded nonwovens, a mixed culture, in equal volumetric ratios, was prepared as described above.

### 2.5. Assessment of the Survival of Microorganisms on Dust-Loaded Nonwovens 

Nonwovens containing different levels of dust were used to assess the survival of the selected microorganism. Dust-loaded nonwovens of an area of 4 cm^2^ were cut from the previously prepared samples and 100 μL of inocula were applied to each sample. The samples were then placed in sterile Petri dishes and incubated in a climatic chamber (Binder-720, Tuttlingen, Germany) at 30 ± 2 °C and relative humidity of 80% for 8 h. The incubation time of the tests corresponded to the use of an FFR during one work-shift.

We used the static quantitative method from the AATCC 100-2004 ‘Antimicrobial Finishes of Textile Materials’ [18], to determine microorganism survival. Samples of nonwovens were tested immediately after inoculation (at t = 0 h) and after 8 h of incubation. The nonwovens were placed in plastic containers with 50 ml of sterile 0.85% saline and shaken for 5 min. Next, serial dilutions of the samples were made in 0.85% saline (from 10^−2^ to 10^−6^), and 1 ml or 0.1 ml of the appropriate dilutions was placed onto sterile Petri dishes, covered with TSA semi-solid medium (Tryptic Soy Agar, Merck, Darmstadt, Germany) for bacteria and MEA (Malt Extract Agar, Merck, Darmstadt, Germany) for yeasts and moulds. The plates were incubated at 37 ± 2 °C for 24–48 h (bacteria, yeasts) and at 27 ± 2 °C for 72 h (moulds). Following incubation, colonies were counted (the result is given in CFU/sample). The tests were carried out in three independent repetitions for each variant of nonwoven, dust-type, and dust deposition time (total of 45 samples were obtained for every test variant).

The microbial survival rate (survivability) *N* for the nonwoven tested after 8 h of incubation was calculated according to the formula:

(2) $$N=\frac{N_{t}\;}{N_{0}}\times 100\%$$

where: *N_0_*—the number of microorganisms present on the filtering nonwoven at t = 0 h, *N_t_*—the number of microorganisms present on the filtering nonwoven after 8 h incubation (CFU/sample).

### 2.6. Microscopic Assessment of Biofilms of Dust-Loaded Filtering Fibres

To assess the development of microbial biofilm, we used nonwoven samples, which were subjected to dust for 4 min (as described in Section 2.3) and then inoculated with a mixed culture (Section 2.4). Samples prepared in this way were stored in a climatic chamber (Binder, Tuttlingen, Germany) under relative humidity of 80 ± 2% and at a temperature of 30 ± 2 °C, for a duration of 7 days. Dust-loaded nonwovens without microorganisms and control samples without dust were also investigated. Analysis of biofilm development was carried out using a scanning electron microscope (SEM) (HITACHI SEM SU8010, Hitachi High-technology Corporation, Tokyo, Japan) following prior sputtering with gold (Q150T ES, Quorum Technologies, Lewes, UK).

### 2.7. The Influence of Microbial Growth on Nanoparticles and Submicron Particles Penetration through Filtering Nonwovens

To determine how joint presence of dust and microorganisms may affect the filtration efficiency of FFRs, we decided to measure the penetration index of sodium chloride (NaCl) nanoparticles and submicron particles of MB nonwovens, which are responsible for high-efficiency filtration. The samples were subjected to dust for 4 min (as described in Section 2.3) and then inoculated with a mixed culture (Section 2.4). After inoculation, the samples were stored in a climatic chamber (Binder, Tuttlingen, Germany) under relative humidity of 80 ± 2% and at a temperature of 30 ± 2 °C for 7 days. Experiments were carried out on a test stand consisting of a Constant Flow Atomizer (3076, TSI, Shoreview, MN, USA), air purification system with a flow regulator (3074B, TSI, Shoreview, MN, USA), aerosol dryer (3062, TSI, Shoreview, MN, USA) and neutralizer (3077, TSI, Shoreview, MN, USA), scanning mobility particle sizer with differential mobility analyser (3080, TSI, Shoreview, MN, USA), condensation particle counter (3775, TSI, Shoreview, MN, USA), and pneumatic test chamber.

A dry and neutralised test aerosol containing sodium chloride particles (size range 7–270 nm) was passed at a predetermined flow rate (47.5 L/min) through the filtering nonwoven mounted in a sample holder. Air samples were collected downstream of the sample, and then directed to an electrostatic particle classifier and a condensation particle counter to determine number and size of NaCl particles penetrating through the sample. Each of the results was calculated as the arithmetic mean over three measurements. Number of NaCl particles upstream of the sample was determined after each measurement. Then, penetration index was calculated according to the formula:

(3) $$N=\frac{N_{d}\;}{N_{u}}\times 100\%$$

where: $N_{d}$—number of particles downstream of the sample, $N_{u}$—number of particles upstream of the sample.

### 2.8. Statistical Analysis

Statistical analyses were performed using STATISTICA 13.1 software (Statsoft, Tulsa, OK, USA). Descriptive statistics for all variables of interest were calculated. A *t*-test at the significance level 0.05 was performed to compare microorganism numbers on the nonwovens after inoculation (t = 0 h and after 8 h of incubation. One-way analysis of variance (ANOVA) at the significance level 0.05 was performed to identify statistical differences between microorganism numbers on different types of nonwovens with medium and high dust levels. When statistical differences were detected (*p* < 0.05), mean values were compared using Tukey’s post hoc procedure at the significance level 0.05. 

## 3. Results and Discussion

### 3.1. Deposition of Dust on Filtering Nonwovens

The dust content in the samples varied and it depended on the deposition time and the type of the filtering material (Table 2).

**Figure 1:**
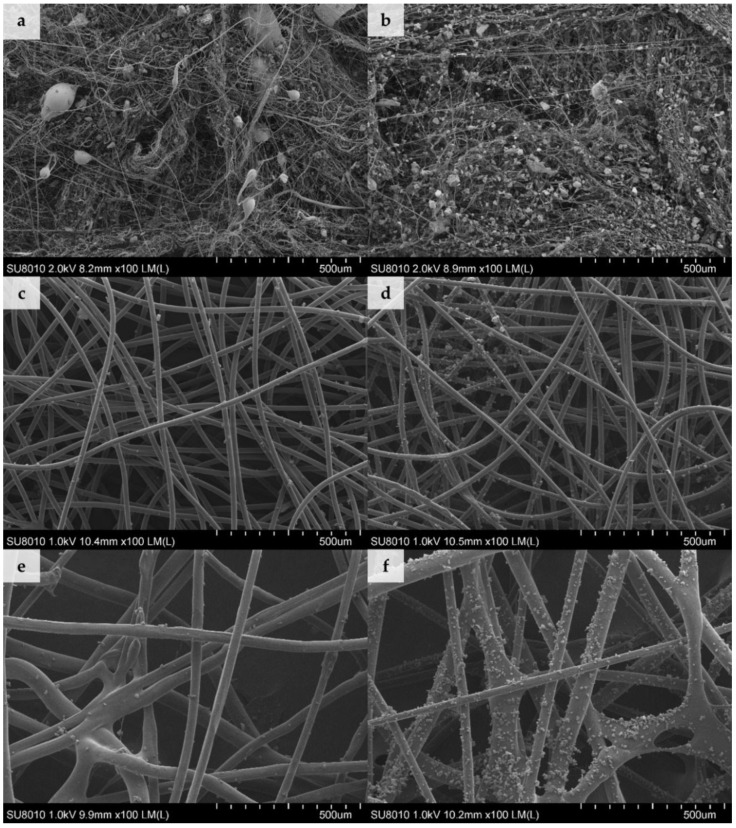
Scanning electron microscopy (SEM) images of nonwovens loaded with dust for 4 min(**a**) MB with dust A, (**b**) MB with dust B, (**c**) SB with dust A, (**d**) SB with dust B, (**e**) NP with dust A, and (**f**) NP with dust B (100× magnification).

Scanning electron microscopy (SEM) was employed to investigate the deposition of dust in the structure of filtering nonwovens (Figure 1).

According to the filtration theory diameters of the elementary fibres, the degree of packing of fibres in the filtering material and the presence of electrostatic charge are among the factors that determine the filtration efficiency of the filtering nonwovens [19]. Our results on the amount of dust deposited in nonwovens (Table 2) are in agreement with those findings. The highest dust contents were observed for densely-packed electret MB nonwovens (Figure 1a–b) with the lowest average fiber diameter of 0.78 ± 0.54 µm (fibre diameter distribution described by a log-normal function). By contrast, the average diameter of the SB nonwoven fibres (Figure 1c–d) was 18.36 ± 0.88 µm (following a normal distribution) and NP nonwoven fibres (Figure 1e–f) was 41.93 ± 13.02 µm (following a bimodal distribution with two well distinguished peaks at 31.53 ± 8.53 µm and 55.62 ± 10.89 µm). In this case the amount of deposited dust was between 55–78% lower than for MB nonwoven, which can be associated with larger diameter of fibers, as well as the lack of electrostatic charges in SB and NP nonwovens.

The average size of dust particles from composting plant was 8.37 ± 5.16 µm and was similar to the one collected from the cement plant (8.92 ± 6.57 µm). The lack of differences between particle sizes was probably due to the fact that both samples were sieved and dried before the experiment, which might have affect their physical sizes. In the case of SB and NP nonwovens, dust agglomerates and individual grains of dimensions ranging from 0.83 µm to 37.50 µm were found to be fixed on the surface of the fibres, regardless of the dust type (Figure 1c–f). On the other hand, dust agglomerates were deposited on MB nonwovens in two ways: smaller grains, of dimensions ranging from 0.42 µm to 0.83 µm, were attached directly to the fibres; while bigger agglomerates, with diameters up to 32.9 µm, were embedded in-between entangled fibres and were also simultaneously attached to several surrounding fibres (Figure 1a–b).

Despite the fact that conditions used for deposition of both types of dust were identical (time and concentration), significant differences in the quantities of dust deposited on the same nonwoven type were observed (Table 2). For type A dust, the increase in sample mass was twice that of type B dust, independent of the nonwoven used. The high density of agglomerates observed in SEM images, with dust from the cement plant, may have resulted from a greater hygroscopicity of cement dust and its effect on deposition of particles on the fibers in the presence of moisture in an air stream [20].

### 3.2. Survival of Microorganisms on Dust-Loaded Filtering Nonwovens

Number of *E. coli* bacteria on all control nonwovens studied at t = 0 h was at a similar level and equalled 7.34 × 10^7^–1.74 × 10^8^ CFU/sample. After 8 h incubation, a greater number of bacteria was noted on all samples (Table 3). The number of *E. coli* bacteria on individual nonwovens in the presence of dust from the composting plant increased from 2.80 × 10^7^–1.34 × 10^8^ CFU/sample at t = 0 h to 3.26 × 10^8^–3.78 × 10^9^ CFU/sample after 8 h. On the other hand, in the presence of dust from the cement plant the number of *E. coli* bacteria was in the range of 5.12 × 10^4^–1.11×10^8^ CFU/sample at t = 0 h and 8.22 × 10^7^–2.19 × 10^9^ CFU/sample after 8 h. Statistically significant differences in the number of *E. coli* bacteria after 8 h (*t*-test) were noted for nonwovens with dust from the composting plant: MB nonwoven (dust concentration of 15.3 mg), SB nonwoven (both concentrations), NP nonwoven (6.3 mg), as well as for NP nonwoven with dust from the cement plant (40.1 mg). In the Tukey test of multiple comparisons of all variants after 8 h, significant differences in the *E. coli* number with respect to the tested variants: f, g, h, i, j, l, n, and o were found for the NP nonwoven control (Table 3).

**Figure 2:**
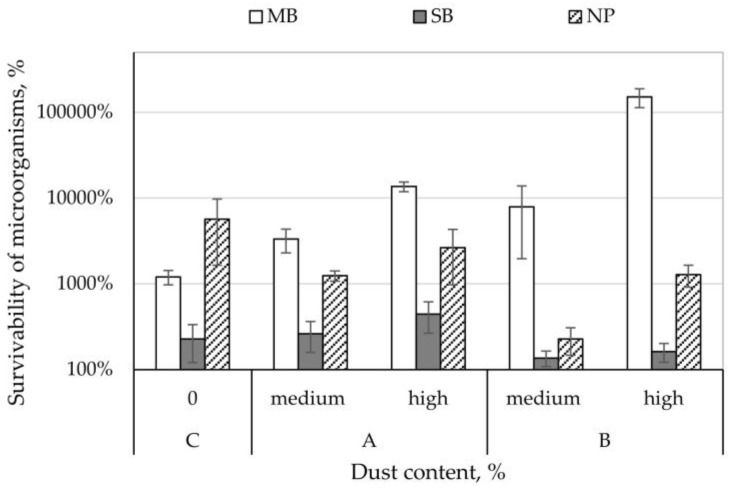
Survivability of *Escherichia coli* bacteria on the filtering nonwovens depending on dust type and content.

Survival rate of *E. coli* bacteria on control nonwovens ranged from 228 to 5692% (Figure 2). On MB nonwoven (in the presence of both dust types) and SB nonwoven (composting plant dust), it increased to the levels of 261–150,946%. In contrast, on NP nonwoven (in the presence of both dust types) and SB nonwoven (cement plant dust), *E. coli* bacteria survivability was lower than on control samples and ranged from 136–2649%. Comparing the increase in survivability on nonwovens between the different dust concentrations, it can be concluded that higher dust concentration from the composting plant increased the survival rate of *E. coli* bacteria by 181–10,333%, while it increased the survival rate by 26–143,040% (Figure 2) in samples from the cement plant.

The number of *C. albicans* on control nonwovens at t = 0 h was at similar levels and equalled 3.02 × 10^5^–5.02×10^5^ CFU/sample. In the presence of dust from the composting plant, it increased to 3.06 × 10^5^–6.80 × 10^5^ CFU/sample at t = 0 h and to 1.94 × 10^6^–2.72 × 10^6^ CFU/sample after 8 h of incubation. By contrast, in the presence of dust from the cement plant, the number of *C. albicans* ranged from 2.66 × 10^5^ to 6.92 × 10^5^ CFU/sample at t = 0 h and 8.38 × 10^4^–1.37 × 10^6^ CFU/sample after 8 h of incubation (Table 4). Statistically significant differences (*t*-test) in *C. albicans* numbers, after 8 h of incubation, were noted for all nonwovens in the presence of dust, except for SB (dust concentration 19.6 mg) and NP (40.1 mg) nonwovens containing dust from the cement plant. Statistically significant differences (Tukey test) in the number of *C. albicans* were found after 8 h for variants: a, b, d, e, g, h, i, j, k, m, n, and o (Table 4).

**Figure 3:**
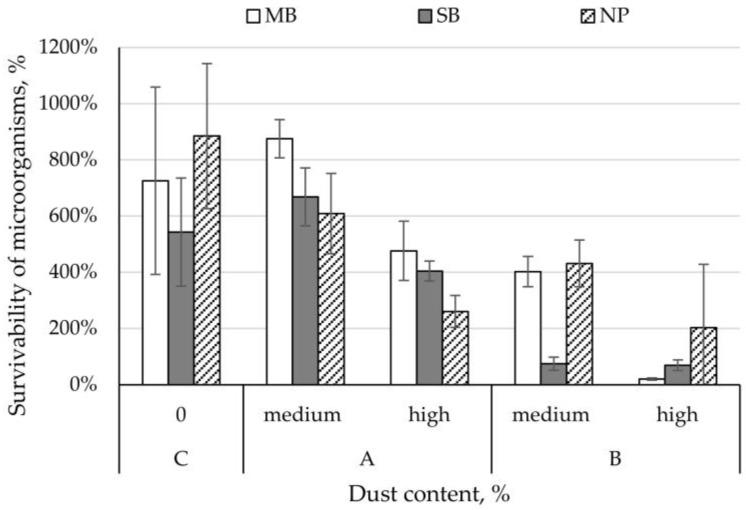
Survivability of *Candida albicans* yeasts on filtering nonwovens depending on dust-type and content.

The survivability of *C. albicans* on control nonwovens ranged from 543% (SB nonwoven) to 885% (NP nonwoven). *C. albicans* survival rate on MB and SB nonwovens with dust from a composting plant with medium concentration was higher than control samples and ranged between 668–875%. In contrast, for the remaining variants of the nonwovens studied, *C. albicans* survivability was lower than control samples and ranged from 20 to 609% (Figure 3). Comparing the increase in survival rate between the different dust concentrations, we found that dust from the composting plant lowered the survival of *C. albicans* by 264% (SB nonwoven), 348% (NP nonwoven), and 399% (MB nonwoven); whilst dust from the cement plant lowered it by 6% (SB nonwoven), 229% (NP nonwoven), and 383% (MB nonwoven) (Figure 3).

The number of *A. niger* on control nonwovens at *t* = 0 h was at similar level and ranged from 2.14 × 10^5^ to 3.26 × 10^5^ CFU/sample. A decrease in *A. niger* numbers was noted for all control samples after 8 h of incubation. The number of *A. niger* on the nonwovens at *t* = 0 h in the presence of various concentrations of dust from the composting plant was 1.44 × 10^5^–2.96 × 10^5^ CFU/sample, and after 8 h 7.12 × 10^4^–3.24 × 10^5^ CFU/sample. In contrast, the number of *A. niger* in the presence of dust from the cement plant ranged from 1.60 × 10^5^ to 5.08 × 10^5^ CFU/sample at *t* = 0 h, and 1.01 × 10^5^–3.60 × 10^5^ CFU/sample after 8 h incubation (Table 5). Statistically significant differences (*t*-test) in the number of *A. niger* fungi after 8 h was noted for MB (35.3 mg), SB (6.0 and 11.7 mg), and NP (6.3 mg) nonwovens with dust from the composting plant, and for NP (12.3 mg) nonwoven with dust from the cement plant. Statistically significant differences (Tukey test) were seen in the number of *A. niger* for SB nonwoven in the presence of dust from the cement plant (13.2 mg) at *t* = 0 h with respect to the remaining experimental variants. After 8 h incubation, significant differences in the number of these moulds were noted with respect to the remaining variants studied: c, d, g, h, i, and l.

**Figure 4:**
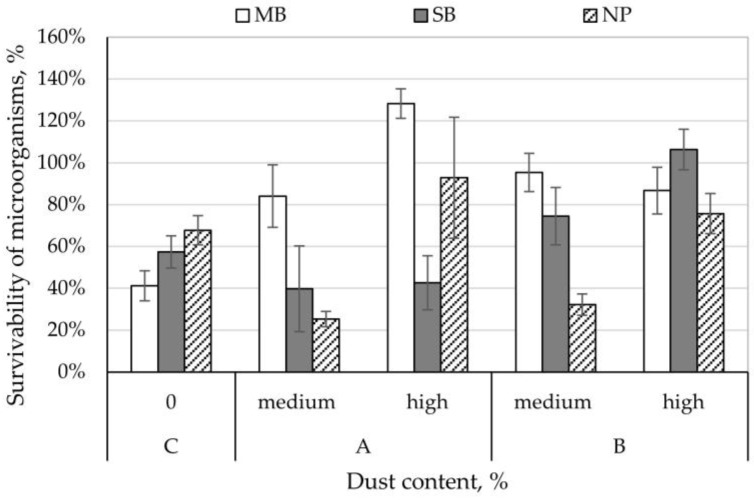
Survivability of *Aspergillus niger* moulds on filtering nonwovens depending on dust-type and content.

The survival rate of *A. niger* on control nonwovens ranged from 41% (MB nonwoven) to 68% (NP nonwoven). The *A. niger* survival rate on SB nonwovens with dust from composting plant with medium and high concentration and NP nonwovens with dust from composting plant and cement plant with medium concentration was lower than for control samples and ranged between 25–43%. In contrast, for the remaining variants of nonwovens studied, *A. niger* survivability was higher than control samples and ranged from 74 to 128% (Figure 4). Comparing the increase in survival rates on nonwovens between the different dust concentrations, it can be stated that dust from the composting plant increases the survivability of *A. niger* by 3–68% and dust from the cement plant increases survival rate by 32–44%; while higher concentration of dust from the cement plant on MB nonwoven lowers *A. niger* survival rate by 8% (Figure 4).

In this paper, we tested the survival of bacteria, yeasts, and moulds on nonwovens used for the construction of filtering half masks. These microorganisms are a health threat in many working environments. Survival, over a time-frame corresponding to a standard working shift (8 h), depended on the type of nonwoven and the species of the microorganism. The highest survival rates were observed for: NP (*N* = 25–5692%) and MB nonwovens (*N* = 20–150,946%), lower for SB nonwoven (*N* = 40–668%). The lower survivability of microorganisms on SB nonwoven can be associated to its low nominal surface mass (20 g/m^2^) and thus lower capacity to collect dust, which constituted a source of nutrients for microorganisms.

The degree of survival of *E. coli*, *C. albicans*, and *A. niger* on fibres in the presence of dust from the composting and cement plants depended not only on the microorganism, but also on the type and amount of dust. In most cases dust from the composting plant (at a concentration of 4.1–14.4%, depending on the nonwoven) and cement plant (3.3–8.5%) increased the survival of *E. coli* bacteria and *A. niger* moulds on the filtering nonwovens. Dust from the composting plant did not increase the survival of *C. albicans*, whereas dust from the cement plant significantly decreased yeast survival. Different groups of microorganisms may be sensitive to biocides in various ways (antiseptics and disinfectants). Based on varied composition of the outer cell layers, it is assumed that Gram-negative bacteria like E. coli are naturally characterized by greater resistance to harmful factors compared to Gram-positive bacteria but smaller than moulds [21]. The survivability (sensitivity) of microorganisms may be affected by many factors that were not the subject of this study, for example, the growth phase of tested microorganisms. However, it should be emphasized that more and more studies indicate not only differences in the sensitivity of individual groups of microorganisms to physical and chemical factors, but also to the occurrence of such differences in another strains of the same species. Collection strains may have different sensitivity from those isolated from the environment, the same can be true for environmental strains but isolated from various places. Such observations were made, among others, during tests of bacteria and fungi strains from ATCC collection (6 strains) and from archives and museums (32) to silver nanoparticles [22], or from ATCC collection (5) and from museums, composting plants, tanneries (18) to nonwovens with Sanitized [23]. The high carbon to nitrogen ratio (C:N = 98.64) in dust from the composting plant promoted growth compared to dust from the cement plant, which had a low ratio (C:N ≈ 10). Gutarowska et al. 2018 found different levels of microbial contamination in sedimented dust samples depending on the workplace—high for samples of cereal dust from a composting plant, lower for dust from a poultry farm and the lowest from a cement plant [7]. The highest number and variety of microorganisms was found in dust from composting plant and in cereal dust, while it was lowest in the case of a cement plant. This confirms that dust from a composting plant is a good source of nutrients that allow the growth of microorganisms in this environment. Moreover, it may provide nutrients that induce the growth of microorganisms (reproduction of bacteria and germination of spores of moulds) on dust-loaded filtering nonwovens, which would explain that, in most cases, more intensive growth of microorganisms was observed on nonwovens loaded with composting plant dust than on those loaded with cement dust. Furthermore, the results obtained by Maus et al. indicate that atmospheric dust present on filtering materials can be a source of nutrients for *A. niger* and with adequate humidity can positively affect the survival of these microorganisms [12]. In model studies, Majchrzycka et al. found that the survival of microorganisms on filtering materials, after 24h incubation at high humidity, correlated with the concentration of dust (9-104% m/m) [15]. In their study they used dust from a combined heat and power plant processing plant biomass. The authors showed that in conditions of high humidity, organic dust on the filtering nonwovens increased the survival of *E. coli* bacteria by 410% in relation to survival of these bacteria on control nonwovens. Likewise, the present study establishes that dust from the composting and cement plants deposited on nonwoven materials for the construction of filtering half-masks increased the survival rate of the tested microorganisms by 149,743% (*E. coli* bacteria), 81% (*A. niger* moulds), and 149% (*C. albicans* yeasts).

### 3.3. Microscopic Assessment of Biofilms on Dust-Loaded Filtering Nonwovens

**Figure 5:**
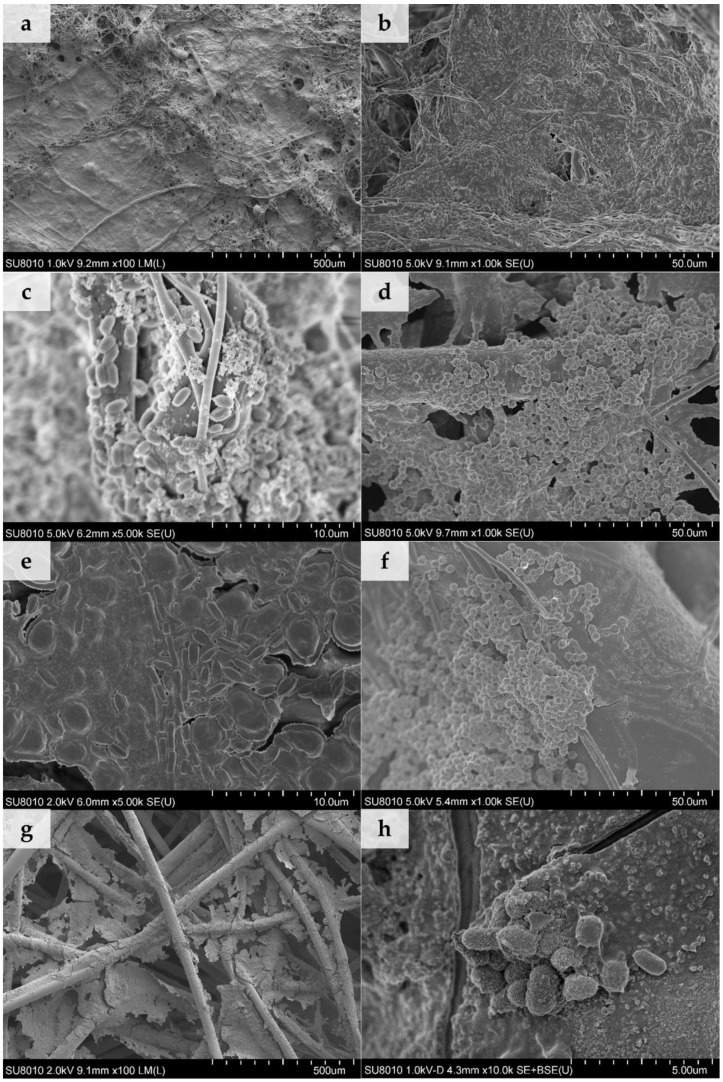
SEM images of biofilms on dust-loaded filtering nonwovens: (**a**) Control MB nonwoven, (**b**) MB with dust A, (**c**) MB with dust B, (**d**) control SB nonwoven, (**e**) SB with dust A, (**f**) control NP nonwoven, (**g**) NP with dust A, and (**h**) NP with dust B.

The ability of microorganisms to grow on nonwovens loaded with different types of dust was also confirmed using a scanning electron microscope (Figure 5).

We determined that on SB and NP nonwovens with no dust, a biofilm did not develop. Clusters of individual microbial cells (spores of *A. niger*), attached to the fibres, are shown in Figure 5d,f. For the MB nonwovens in all tested cases, individual microbial cells (Figure 5c), as well as bacterial biofilm sticking fibres and dust together, were observed (Figure 5a,b). For SB and NP nonwovens loaded with both types of dust a biofilm covering single fibers (Figure 5h,e) and one formed in the spaces between them (Figure 5g) was observed. When biofilm is firmly attached to the fibres it lowers porosity of the nonwoven. Individual fibres sticking together following biofilm formation may cause increased breathing resistance when filtering half masks are being used and limit the penetration of dust particles within the structure of the filtering material. On the other hand, cracked fragments of biofilm (Figure 5g) may detach from the nonwoven surface (especially at high air flow velocities), migrate within the filtering material, and penetrate into the organism during breathing, which would constitute a source of secondary inhalation exposure for the worker.

### 3.4. The Influence of Microbial Growth on Nanoparticles and Submicron Particles Penetration through Filtering Nonwovens

**Figure 6:**
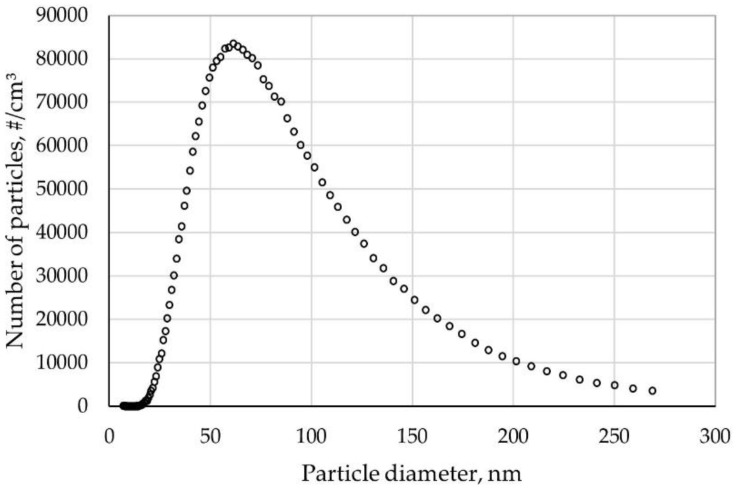
NaCl particle size distribution.

Figure 6 shows the size distribution of NaCl aerosol used for penetration testing. Each data point is averaged over five independent measurements. 

The diameters of NaCl particles followed log-normal distribution. The mean geometric diameter for the particle count distribution was 43.7 nm, and the maximum particle number distribution was 8.35 × 10^4^ (Figure 6).

**Figure 7:**
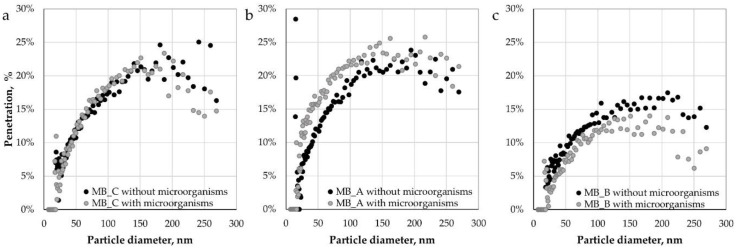
Penetration of nanoparticles and submicron particles through: (**a**) Control MB nonwovens, (**b**) MB nonwovens with dust A, and (**c**) MB nonwovens with dust B.

Figure 7 shows penetration of nanoparticles and submicron particles as a function of particle size (7–270 nm) for pristine and inoculated MB nonwovens both with and without dust.

Similar penetrations (up to approx. 25%) were observed for control MB nonwovens and MB nonwovens containing dust A (both with and without microorganisms). Significantly lower values of penetration were measured for MB nonwovens with dust B (up to 17.5%), which might be due to higher dust content that blocks part of the pores of the filtering materials. The obtained penetration values (for control samples) are higher than previously reported in the literature [24], which may be the result of the applied disinfection process in isopropyl alcohol vapours that could lead to a partial discharge of electret material, and hence the weakening of the electrostatic interaction between the fibres and the aerosol particles. This observation is consistent with the previous findings showing diminishing effect of isopropyl alcohol treatment on the electret performance of nonwoven filters [25,26,27]. For control nonwovens, no effect of microbial growth on penetration was observed, while for nonwovens loaded with dust A, the differences occurred only in the particle size range of 20-110 nm. This may indicate the influence of biofilms formation in the presence of dust from composting plant (type A) on the effectiveness of dominant mechanisms in the case of filtration of nanoparticles by such nonwovens, i.e., diffusion mechanism [28,29,30]. Noticeable decreases in penetration (up to 8.9%) in the whole particle size range as a result of microbial growth were noted for the MB nonwoven loaded with dust B. This difference may result from better conditions for biofilm formation, which, by gluing the fibers, reduced the porosity of the nonwoven, and thus increased the efficiency of capturing particles from the stream of flowing air.

## 5. Conclusions

All tested microorganisms, *E. coli*, *C. albicans*, and *A. niger*, were able to survive on dust-loaded polypropylene filtering nonwovens. Some nonwovens were much better suited for survival than others. The microorganisms survived best on the NP nonwoven (survival rate *N* = 68–5692%) and MB nonwoven (*N* = 41–1203%), and had lower survivability on SB nonwoven (*N* = 57–543%). Survival was the highest for *E. coli* (*N* = 136–150,946%), lower for *C. albicans* (*N* = 20–885%), and lowest for *A. niger* (*N* = 25–128%). The degree of survival of *E. coli*, *C. albicans*, and *A. niger* on the fibres in the presence of dust from the composting or cement plant, depended on the type of microorganism and the amount of dust. Higher survival of *E. coli* bacteria and *A. niger* moulds was detected with increasing dust concentrations. The presence of dust did not increase the survival of *C. albicans* yeasts, instead dust from the cement plant significantly reduced the survival of yeasts. Development of microbiological biofilms on tested nonwovens was also confirmed, which may influence filtration efficiency and constitute a source of secondary exposure. Joint influence of dust and microbial growth on penetration of nanoparticles and submicron particles was observed (increase of penetration in case of nonwovens with dust A and decrease for dust B). Although penetration tests were conducted on a single layer of the FFR and not a complete device, and the electret performance was diminished with the use of isopropyl alcohol, the results indicate that both of those factors should be considered during selection of respirators used for protection against airborne biological agents and establishing times that they can be safely used by workers. 

## Figures and Tables

**Table 1:** Characteristics of filtering nonwovens.

Nonwoven Type Designation	Nonwoven Type	Function in FFR Structure	Nominal Surface Mass, G/M^2^
MB	melt-blown, electret	high-efficiency filtration	90
SB	spun-bonded	pre-filtration of coarse dust particles	20
NP	needle-punched, calandered	stiffening of the FFR structure	110

**Table 2:** Dust content in the nonwoven samples.

Nonwoven Type	Dust Type	Mass of Dust Deposited in the Nonwoven (Deposition Time), Mg/ Dust Content in the Filtering Nonwoven, %	
		Medium (2 min)	High (4 min)
MB	A	15.3/5.9	35.3/13.5
	B	37.9/2.0	89.2/5.5
SB	A	6.0/9.6	11.7/14.4
	B	13.2/4.8	19.6/8.5
NP	A	6.3/1.6	29.0/4.1
	B	12.3/0.8	40.1/3.3

A—dust from a composting plant; B—dust collected in the cement plant.

**Table 3:** Number of *Escherichia coli* bacteria on the filtering nonwovens depending on dust type and content.

Nonwoven Type	Dust Type	Dust Content	Variant	Number of Microorganisms at 0 h, CFU/Sample	Number of Microorganisms at 8 h, CFU/Sample
MB	C	0	a	M: 9.26 × 10^7^ SD: 5.29 × 10^7^	M: 1.17 × 10^9#^ SD: 7.86 × 10^8^
	A	medium	b	M: 7.70 × 10^7^ SD: 7.60 × 10^7^	M: 1.97 × 10^9#^ SD: 9.59 × 10^8^
		high	c	M: 2.80 × 10^7^ SD: 1.73 × 10^7^	M: 3.78 × 10^9#^ SD: 2.09 × 10^9^
	B	medium	d	M: 2.12 × 10^7^ SD: 1.16 × 10^7^	M: 2.19 × 10^9^ SD: 2.57 × 10^9^
		high	e	M: 5.12 × 10^4f^ SD: 3.99 × 10^4^	M: 8.22 ×10^7k^ SD: 8.51 × 10^7^
SB	C	0	f	M: 1.74 × 10^8e^ SD: 1.51 × 10^8^	M: 3.92 × 10^8k^ SD: 3.13 × 10^8^
	A	medium	g	M: 1.34 × 10^8^ SD: 8.51 × 10^7^	M: 3.26 × 10^8#k^ SD: 1.62 × 10^8^
		high	h	M: 1.30 × 10^8^ SD: 9.41 × 10^7^	M: 4.38 × 10^8#k^ SD: 6.18 × 10^7^
	B	medium	i	M: 1.11 × 10^8^ SD: 5.05 × 10^7^	M: 1.46 × 10^8k^ SD: 5.52 × 10^7^
		high	j	M: 1.05 × 10^8^ SD: 7.30 × 10^7^	M: 1.46 × 10^8k^ SD: 7.10 × 10^7^
NP	C	0	k	M: 7.34 × 10^7^ SD: 3.15 × 10^7^	M: 5.04 × 10^9f,g,h,i,j,l,n,o^ SD: 5.00 × 10^9^
	A	medium	l	M: 7.04 × 10^7^ SD: 5.66 × 10^7^	M: 8.22 × 10^8#k^ SD: 5.82 × 10^8^
		high	m	M: 1.31 × 10^8^ SD: 1.28 × 10^8^	M: 3.72 × 10^9^ SD: 3.60 × 10^9^
	B	medium	n	M: 8.96 × 10^7^ SD: 6.92 × 10^7^	M: 1.58 × 10^8k^ SD: 3.11 × 10^7^
		high	o	M: 1.58 × 10^7a^ SD: 1.15 × 10^7^	M: 1.79 × 10^8#k^ SD: 1.17 × 10^8^

C—control; A—dust from a composting plant; B—dust collected in the cement plant; M—mean value, SD—standard deviation, #—statistically significant differences between bacteria number at t = 0 h and after 8 h of incubation for all variants; (*t*-test, α = 0.05); a–o in the upper index—the variants, for which statistically significant differences between microorganism numbers on nonwovens with different dust content were found (Tukey test, α = 0.05).

**Table 4:** Number of *Candida albicans* on filtering nonwovens depending on dust type and content.

Nonwoven Type	Dust Type	Dust Content	Variant	Number of Microorganisms at 0 h, CFU/Sample	Number of Microorganisms at 8 h, CFU/Sample
MB	C	0	a	M: 5.02 × 10^5^ SD: 5.93 × 10^4^	M: 3.82 × 10^6#d,e,i,j,m,n,o^ SD: 2.49 × 10^6^
	A	medium	b	M: 3.14 × 10^5^ SD: 5.37 × 10^4^	M: 2.72 × 10^6#e,j^ SD: 2.77 × 10^5^
		high	c	M: 4.00 × 10^5^ SD: 5.34 × 10^4^	M: 1.94 × 10^6#^ SD: 7.02 × 10^5^
	B	medium	d	M: 2.66 × 10^5k,i^ SD: 1.15 × 10^5^	M: 1.03 × 10^6#a^ SD: 2.93 × 10^5^
		high	e	M: 4.06 × 10^5^ SD: 1.07 × 10^5^	M: 8.38 × 10^4#a,b,g,h,k^ SD: 3.04 × 10^4^
SB	C	0	f	M: 4.18 × 10^5^ SD: 1.70 × 10^5^	M: 2.08 × 10^6#^ SD: 1.03 × 10^6^
	A	medium	g	M: 4.18 × 10^5^ SD: 2.29 × 10^5^	M: 2.72 × 10^6# e,j^ SD: 1.26 × 10^6^
		high	h	M: 6.80 × 10^5d^ SD: 4.35 × 10^5^	M: 2.66 × 10^6#e^ SD: 1.45 × 10^6^
	B	medium	i	M: 6.92 × 10^5d,k,m,n^ SD: 2.80 × 10^5^	M: 5.38 × 10^5#a^ SD: 3.08 × 10^5^
		high	j	M: 4.02 × 10^5^ SD: 1.04 × 10^5^	M: 2.86 × 10^5a,b,g,k^ SD: 1.49 × 10^5^
NP	C	0	k	M: 3.02 × 10^5i,d^ SD: 1.06 × 10^5^	M: 2.88 × 10^6#e,j^ SD: 1.67 × 10^6^
	A	medium	l	M: 3.76 × 10^5^ SD: 1.22 × 10^5^	M: 2.28 × 10^6#^ SD: 1.07 × 10^6^
		high	m	M: 3.06 × 10^5i^ SD: 6.47 × 10^4^	M: 8.18 × 10^5#a^ SD: 3.26 × 10^5^
	B	medium	n	M: 3.10 × 10^5i^ SD: 5.57 × 10^4^	M: 1.37 × 10^6#a^ SD: 4.65 × 10^5^
		high	o	M: 3.94 × 10^5^ SD: 6.50 × 10^4^	M: 9.10 × 10^5a^ SD: 1.28 × 10^6^

C—control; A—dust from a composting plant; B—dust collected in the cement plant; M— mean value, SD—standard deviation, #—statistically significant differences between bacteria number at *t* = 0 h and after 8 h of incubation for all variants; (*t*-test, α = 0.05); a–o in the upper index—the variants, for which statistically significant differences between microorganisms numbers on nonwovens with different dust content were found (Tukey test, α = 0.05).

**Table 5:** Number of *Aspergillus niger* moulds on the filtering nonwovens depending on dust-type and content.

Nonwoven Type	Dust Type	Dust Content	Variant	Number of Microorganisms at 0 h, CFU/Sample	Number of Microorganisms at 8 h, CFU/Sample
MB	C	0	a	M: 3.26 × 10^5^ SD: 1.00 × 10^5^	M: 1.38 × 10^5#c,i^ SD: 4.44 × 10^4^
	A	medium	b	M: 2.40 × 10^5i^ SD: 5.87 × 10^4^	M: 1.88 × 10^5ci^ SD: 1.64 × 10^4^
		high	c	M: 2.54 × 10^5i^ SD: 3.21 × 10^4^	M: 3.24 × 10^5#a,b,e-h,j-o^ SD: 2.61 × 10^4^
	B	medium	d	M: 2.42 × 10^5i^ SD: 3.77 × 10^4^	M: 2.32 × 10^5 g-i, l,n^ SD: 4.87 × 10^4^
		high	e	M: 2.22 × 10^5i^ SD: 6.18 × 10^4^	M: 1.90 × 10^5c,i,l^ SD: 4.85 × 10^4^
SB	C	0	f	M: 2.28 × 10^5i^ SD: 5.17 × 10^4^	M: 1.30 × 10^5#c,i^ SD: 3.32 × 10^4^
	A	medium	g	M: 2.24 × 10^5i^ SD: 7.70 × 10^4^	M: 9.70 × 10^4#c,d,i^ SD: 8.34 × 10^4^
		high	h	M: 2.36 × 10^5i^ SD: 7.44 × 10^4^	M: 1.07 × 10^5# c,d,i^ SD: 5.59 × 10^4^
	B	medium	i	M: 5.08 × 10^5b-o^ SD: 1.84 × 10^5^	M: 3.60 × 10^5a,b,d-o^ SD: 1.09 × 10^5^
		high	j	M: 1.64 × 10^5^ SD: 4.16 × 10^4^	M: 1.72 × 10^5^ SD: 3.56 × 10^4^
NP	C	0	k	M: 2.14 × 10^5i^ SD: 7.86 × 10^4^	M: 1.47 × 10^5c,i^ SD: 6.51 × 10^4^
	A	medium	l	M: 2.96 × 10^5i^ SD: 1.16 × 10^5^	M: 7.12 × 10^4#c-e,i^ SD: 1.17 × 10^4^
		high	m	M: 1.44 × 10^5i^ SD: 7.09 × 10^4^	M: 1.25 × 10^5c,i^ SD: 4.19 × 10^4^
	B	medium	n	M: 3.02 × 10^5i^ SD: 9.42 × 10^4^	M: 1.01 × 10^5#c,d,i^ SD: 4.71 × 10^4^
		high	o	M: 1.60 × 10^5i^ SD: 2.92 × 10^4^	M: 1.23 × 10^5c,i^ SD: 3.50 × 10^4^

C—control; A—dust from a composting plant; B—dust collected in the cement plant; M—mean value, SD—standard deviation, #—statistically significant differences between bacteria number at *t* = 0 h and after 8 h of incubation for all variants; (*t*-test, α =0.05); a-o in the upper index - the variants, for which statistically significant differences between microorganisms numbers on nonwovens with different dust content were found (Tukey test, α = 0.05).

## References

[1] Directive 2000/54/EC of The European Parliament and of The Council of 18 September 2000 on the Protection of Workers From Risks Related to Exposure to Biological Agents at Work. http://www.biosafety.be/PDF/2000_54.pdf.

[2] Viegas S., Caetano L.A., Korkalainen M., Faria T., Pacífico C., Carolino E., Quintal Gomes A., Viegas C. (2017). Cytotoxic and inflammatory potential of air samples from occupational settings with exposure to organic dust. Toxics.

[3] Rusca S., Charrière N., Droz P.O., Oppliger A. (2008). Effects of bioaerosol exposure on work-related symptoms among Swiss sawmill workers. Int. Arch. Occup. Environ. Health.

[4] Matheson M., Benke G., Raven J., Sim M., Kromhout H., Vermeulen R., Johns D.P., Walters E.H., Abramson M.J. (2005). Biological dust exposure in the workplace is a risk factor for chronic obstructive pulmonary disease. Thorax.

[5] Ahmed H.O., Abdullah A.A. (2012). Dust exposure and respiratory symptoms among cement factory workers in the United Arab Emirates. Ind. Health.

[6] Pearson C., Littlewood E., Douglas P., Robertson S., Gant T.W., Hansell A.L. (2015). Exposures and health outcomes in relation to bioaerosol emissions from composting facilities: A systematic review of occupational and community studies. J. Toxicol. Environ. Heal. Part B.

[7] Gutarowska B., Szulc J., Nowak A., Otlewska A., Okrasa M., Jachowicz A., Majchrzycka K. (2018). Dust at various workplaces—Microbiological and toxicological threats. Int. J. Environ. Res. Public Health.

[8] Eduard W., Heederik D., Duchaine C., Green B.J. (2012). Bioaerosol exposure assessment in the workplace: The past, present and recent advances. J. Environ. Monit..

[9] Dutkiewicz J., Śpiewak R., Jabłoński L., Szymańska J. (2007). Biological Occupational Risk Factors. Classification, Exposed Occupational Groups, Measurement, Prevention (in Polish).

[10] Directive 89/656/EEC of 30 November 1989 on the Minimum Health and Safety Requirements for the Use by Workers of Personal Protective Equipment at the Workplace. https://eur-lex.europa.eu/legal-content/EN/TXT/PDF/?uri=CELEX:31989L0656&from=en.

[11] Majchrzycka K., Okrasa M., Skóra J., Gutarowska B. (2016). Evaluation of the survivability of microorganisms deposited on filtering respiratory protective devices under varying conditions of humidity. Int. J. Environ. Res. Public Health.

[12] Maus R., Goppelsröder A., Umhauer H. (2001). Survival of bacterial and mold spores in air filter media. Atmos. Environ..

[13] Jankowska E., Reponen T., Willeke K., Grinshpun S.A., Choi K.J. (2000). Collection of fungal spores on air filters and spore reentrainment from filters into air. J. Aerosol. Sci..

[14] Szulc J., Otlewska A., Okrasa M., Majchrzycka K., Sulyok M., Gutarowska B. (2017). Microbiological contamination at workplaces in a combined heat and power (CHP) station processing plant biomass. Int. J. Environ. Res. Public Health.

[15] Majchrzycka K., Okrasa M., Szulc J., Gutarowska B. (2017). The impact of dust in filter materials of respiratory protective devices on the microorganisms viability. Int. J. Ind. Ergon..

[16] GESTIS: International Occupational Exposure Limit Values for Chemical Agents. http://limitvalue.ifa.dguv.de/.

[17] Schuster E., Dunn-Coleman N., Frisvad J., van Dijck P. (2002). On the safety of Aspergillus niger—A review. Appl. Microbiol. Biotechnol..

[18] (2004). AATCC Test Method 100-2004. Antibacterial Finishes on Textile Materials: Assessment of Antibacterial Finishes on Textile Materials. Technical Manual/2010. http://www.manufacturingsolutionscenter.org/aatcc-100-antibacterial-finishes-textile.html.

[19] Brown R.C. (1993). Air filtration: An Integrated Approach to the Theory and Applications of Fibrous Filters.

[20] Miguel A.F. (2003). Effect of air humidity on the evolution of permeability and performance of a fibrous filter during loading with hygroscopic and non-hygroscopic particles. J. Aerosol Sci..

[21] Russell A.D. (2003). Similarities and differences in the responses of microorganisms to biocides. J. Antimicrob. Chemother..

[22] Gutarowska B., Skóra J., Zduniak K., Rembisz D. (2012). Analysis of the sensitivity of microorganisms contaminating museums and archives to silver nanoparticles. Int. Biodeterior. Biodegradation.

[23] Gutarowska B., Skóra J., Nowak E., Łysiak I., Wdówka M. (2014). Antimicrobial activity and filtration effectiveness of nonwovens with Sanitized for respiratory protective equipment. Fibres Text. East. Eur..

[24] Brochocka A., Makowski K., Majchrzycka K., Grzybowski P. (2013). Efficiency of filtering materials used in respiratory protective devices against nanoparticles. Int. J. Occup. Saf. Ergon..

[25] Viscusi D.J., Bergman M.S., Eimer B.C., Shaffer R.E. (2009). Evaluation of five decontamination methods for filtering facepiece respirators. Ann. Occup. Hyg..

[26] Viscusi D., King W.P., Shaffer R.E. (2007). Effect of decontamination on the filtration efficiency of two filtering facepiece respirator models. J. Int. Soc. Respir. Prot..

[27] Zhou Y., Cheng Y.S. (2016). Evaluation of N95 filtering facepiece respirators challenged with engineered nanoparticles. Aerosol Air Qual. Res..

[28] Bałazy A., Podgórski A., Gradoń L. (2004). Filtration of nanosized aerosol particles in fibrous filters. I—Experimental results. J. Aerosol Sci..

[29] Wang C., Otani Y. (2013). Removal of nanoparticles from gas streams by fibrous filters: A review. Ind. Eng. Chem. Res..

[30] Wang J., Chen D.R., Pui D.Y.H. (2007). Modeling of filtration efficiency of nanoparticles in standard filter media. J. Nanoparticle Res..

